# Transcript Polymorphism Rates in Soybean Seed Tissue Are Increased in a Single Transformant of* Glycine max*

**DOI:** 10.1155/2016/1562041

**Published:** 2016-11-29

**Authors:** Kevin C. Lambirth, Adam M. Whaley, Jessica A. Schlueter, Kenneth J. Piller, Kenneth L. Bost

**Affiliations:** ^1^Department of Bioinformatics and Genomics, University of North Carolina at Charlotte, North Carolina Research Campus, Kannapolis, NC 28081, USA; ^2^Department of Bioinformatics and Genomics, University of North Carolina at Charlotte, Charlotte, NC 28223, USA; ^3^Department of Biological Sciences, University of North Carolina at Charlotte, Charlotte, NC 28223, USA

## Abstract

Transgenic crops have been utilized for decades to enhance agriculture and more recently have been applied as bioreactors for manufacturing pharmaceuticals. Recently, we investigated the gene expression profiles of several in-house transgenic soybean events, finding one transformant group to be consistently different from our controls. In the present study, we examined polymorphisms and sequence variations in the exomes of the same transgenic soybean events. We found that the previously dissimilar soybean line also exhibited markedly increased levels of polymorphisms within mRNA transcripts from seed tissue, many of which are classified as gene expression modifiers. The results from this work will direct future investigations to examine novel SNPs controlling traits of great interest for breeding and improving transgenic soybean crops. Further, this study marks the first work to investigate SNP rates in transgenic soybean seed tissues and demonstrates that while transgenesis may induce abundant unanticipated changes in gene expression and nucleotide variation, phenotypes and overall health of the plants examined remained unaltered.

## 1. Introduction

Over the past three decades, transgenic plant biotechnology has become integrated into the agriculture of nearly all societies worldwide. Genetically modified food crops have revolutionized conventional farming methods by increasing yields [1], bolstering resistance to pests and herbicides [2, 3], and enhancing resistance to environmental stresses [4]. Furthermore, plant systems have been employed for cost-effective production of biological therapeutics such as oral vaccine candidates [5], monoclonal antibody production [6], and accumulation of therapeutic and diagnostic proteins [7, 8]. Coupled with self-regenerating properties and the natural ability to stably store proteins in varied environmental conditions, plants offer a multitude of advantages over traditional cell culture systems for protein generation [9]. Over the past 10 years, our laboratory has focused on* Glycine max* as a model system for the generation and storage of recombinant proteins that are currently expensive to manufacture and difficult to procure or synthesize in bacterial and cell-based cultures and as oral vaccine candidates by targeting gut-associated lymphoid tissues (GALT) for immune stimulation or suppression [5, 8, 10–13]. Advantages for using soybean as an expression system include high protein content and yield, self-fertilization to simplify the generation of homozygous transformants, and the stability of endogenous proteins in seed tissues, which we have reviewed in depth previously [14].

Selective farming of agricultural food sources has been conducted to propagate advantageous phenotypes such as improved germplasm size and quantity, particularly in crop plants of maize, soybean, rice, cereals, and potato [15]. Backcrossing of parents allows for further control over traits seen in progeny, although this process is time consuming and often unpredictable, particularly in species that are not self-pollinating. Segregation of alleles does not always produce desired functionality in overall phenotype, and due to individual variability, it may come at the cost of overall fitness [16].

*Glycine max,* or the modern cultivated soybean, is a legume branched from the wild species* Glycine soja* and is classified as a diploidized tetraploid (2*n* = 40). Soybean has a relatively lengthy growth cycle (~6 months) and is primarily cultivated for its seed, which is high in both protein and oil content [17]. It is also primarily a self-pollinating dicot, as the anthers and stigma mature together in the same flower of most cultivars preventing cross-pollination with other plants [18]. This makes soybean desirable for several reasons: The first one is the simplicity of inheritance selections when breeding, and the other is the high protein content (~40% by weight) of the soybean seed itself, which through millions of years of evolution has been designed to maintain the durability and stability of its internal cargo until optimum conditions are met for germination. Soluble protein extraction from soy seed is also straightforward and has proven to be efficient for the generation and purification of vaccine antigens, therapeutic proteins, and diagnostic peptides [7–9]. This makes soybean an ideal candidate platform as a bioreactor for the generation, accumulation, and long-term storage of plant-based biologics [14], removing the trade-off dichotomy of either high yield (leaf tissue expression) or long-term stability (seed tissue).

Single nucleotide polymorphisms (SNPs) and insertions/deletions (INDELS) are major driving forces behind species evolution and diversity, either changing a single nucleotide base in the former or shifting the transcriptional reading frame in the latter. The end results, depending on the position of the base changes, may alter protein products controlling certain traits. SNPs may result in benign variations in humans such as eye color, skin color [19], and finger length changes [20] or alternatively may be responsible for debilitating disorders such as lung cancer and multiple sclerosis [21]. INDELS and nonsense polymorphisms can have large effects on gene products, disrupting transcription start sites and essential cellular processes. Soybean has experienced a dramatic reduction in genomic variability following the development of worldwide cultivation practices of the wild ancestor* Glycine soja*, all but eliminating rare allelic combinations in domesticated varieties [22]. Due to this long-term domestication,* Glycine max* shares only 97.65% genomic sequence similarity with* Glycine soja* and contains 425 novel genes that are not present in the wild cultivar that directly control seed development, oil content, and protein concentration [23].

Recently, we investigated the pleiotropic effects on the transcriptome of soybean seed tissues expressing and accumulating high levels of recombinant protein [24]. Results revealed no correlation between transgene or recombinant protein expression level and the quantity of differentially regulated genes, although one of the transgenic lines contained over 3000 differentially expressed genes. We concluded that the gene expression differences observed may have been due to the specific properties of the recombinant proteins themselves on the homeostatic environment of the seed, or due to random mutagenesis; however, molecular characterizations of transcript structure and internal modifications remained unknown. Given the ability of single base mutations to cause severe effectual repercussions, in conjunction with the high number of differentially expressed genes we previously detected in our transgenic soybean line, we explored whether transcript polymorphism rates were influenced in the same manner.

In order to elucidate possible transcript modifications as a result of transformation, we utilized the publicly available RNA-seq datasets generated by our previous transcriptome sequencing study [24] to assess exome INDELs and SNP rates in all of the transgenic samples investigated previously. This allowed us to examine gene variances exclusive to the transgenic plants and to characterize the possible resulting effects of these polymorphisms in conjunction with the differentially expressed genes we previously characterized. To our knowledge, this is the first effort to characterize exome polymorphisms in transgenic soybean seed tissues and will serve to further interpret alterations and their possible effects resulting from transgenesis.

## 2. Materials and Methods

In this study, we utilized datasets obtained from whole-transcriptome sequencing of transgenic soybean seed tissues expressing three different transgenes to detect exomic polymorphisms. Each experimental group consisted of nine seeds from each of the three transgenic events (designated ST111, ST77, and 764) as well as the wild type controls. All transgenic plants were generated using* Agrobacterium* transformation and taken to homozygosity over multiple generations prior to sequence acquisition. The transgenes and recombinant proteins expressed and accumulated in the seed tissues have been described previously by our lab [24]. Quality control filtering and assembly were conducted as part of the previous study prior to polymorphism detection and annotation with samtools and bcftools.

### 2.1. Exon SNP/INDEL Analysis

Soybean cultivation, RNA extraction, and differential gene expression analyses were carried out using RNA-seq as previously described in Lambirth et al. [24]. In order to facilitate exome SNP calls, samtools version 1.2 [25, 26] was used to index the soybean reference genome version 2.75 sequence file obtained from Phytozome [27], which was amended to contain scaffolds of all three T-DNA sequences using the -*faidx* command to allow alignment of nonreference transgene transcripts. Alignment files previously generated by TopHat [28] for all previously reported samples [24] in  .bam format were converted to  .bcf files using the* -mpileup* command with* -g* and* -f* parameters to specify the output format and to use the indexed reference fasta file. The bcftools* call* command was subsequently used on the indexed  .bcf files with the* -c* parameter to invoke the original consensus calling method enabling SNP and INDEL identification. The* stat* and* plot-vcfstats* commands were used to generate statistical summaries for each sample. Total SNP counts for each sample were averaged to calculate the standard deviation and standard error for each experimental group of wild type and transgenic seeds, and unpaired two-tailed *t*-tests were used to compare each transgenic group with wild type. Individual nucleotide base changes, transition and transversion rates, INDELS, and single and multiallele SNPs were also recorded and compared between groups. To identify SNPs present in each experimental group, the intersections of each variant file for all nine individual replicates in the ST77, ST111, 764, and WT groups were taken. SNPs in these combined files overlapping between all three transgenic groups, as well as those unique to each event when compared to WT, were pulled from the  .vcf files using the -*vcf-isec* command in vcftools [29]. SNPs shared between the transgenics and WT control group were removed to generate  .vcf files containing SNPs found exclusively in each transformation event.

Visualization of SNPs and INDELS was conducted using Circos version 0.69-2 [30]. Variance files in  .bcf format from the samtools/SNPeff output were converted to  .vcf files with the* -bcftools view*-*o* command; then, using the* cut -f 1,2,6* command, base calls, quality score metrics, and chromosomal locations were extracted. Formatted tab delimited files were converted to the input text file for Circos using an in-house script generated in Python (version 2.7.11), which filtered the calls to exclude all polymorphisms with quality scores below 15. The script is included in the Additional Files of this manuscript (in Supplementary Material available online at http://dx.doi.org/10.1155/2016/1562041). Plots were then generated using the -*circos -conf circus.conf* command to display SNP distributions of the transgenic and wild type samples for all 20 soybean genomic chromosomes. SNPs were spaced in the plots every 20 bases and layered vertically.

To predict any possible translational effects resulting from detected SNPs and INDELS, snpEff version 4.1i [31] was utilized on the resulting variance call files generated by bcftools. A custom database for snpEff was constructed using the -*build* command consisting of the soybean genome FASTA reference file described previously containing our T-DNA sequences, as well as the gene model  .gff3 file from the Cufflinks output from our previous RNA-seq data [24] obtained from Phytozome. The  .gff3 file provided a reference index for gene positions and identifiers, as well as intron/exon models and untranslated regions. No codon table configuration was necessary as* Glycine max* utilizes standard codon triplets allowing snpEff to run with the default parameters, employing the SNP/INDEL call  .vcf file from bcftools as input. Multithreaded processing using the* -t* option was not used, as this removes statistical calculations and the resulting reports from the output file. Statistical comparisons of SNP rates and effects between each group were conducted using two-tailed unpaired *t*-tests in Microsoft Excel. Functional annotation of genes containing variants was accomplished by loading the gene output list from snpEff into the agricultural gene ontology (GO) enrichment tool AgriGO [32] using the integrated single enrichment analysis tool with the* Glycine max* Wm82.a2.v1 background reference. Significant terms were calculated using Fisher's exact test with a corrected false discovery rate *p* value threshold of 0.05. All computations were conducted on an Apple Macbook Pro with a 2.7 GHz quad-core Intel i7 processor and 16 GB of DDR3 RAM.

## 3. Results

### 3.1. SNP Rates in Transgenic Soybean Exons

**Figure 1:**
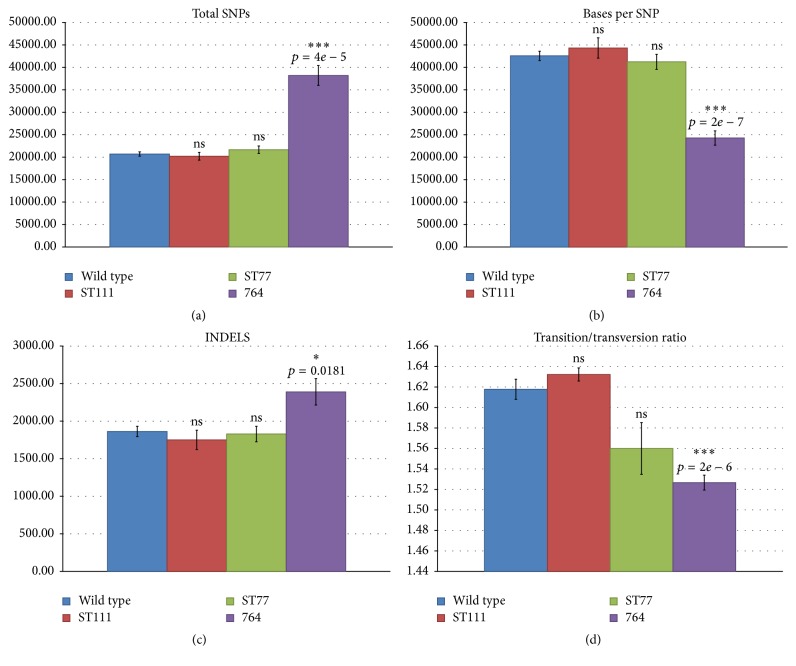
Summary of SNP and INDEL counts. Means of total polymorphisms (a), polymorphism rates (b), insertions/deletions (c), and transition to transversion ratios (d) were calculated all four experimental groups. Standard error bars as well as statistical results from unpaired *t-*tests between each group and wild type are shown. Groups marked with an asterisk indicate significance (*p* < 0.05 = *∗*; *p* < 0.001 = *∗∗∗*). Wild type is shown in blue, ST111 in red, ST77 in green, and 764 in purple.

**Figure 2:**
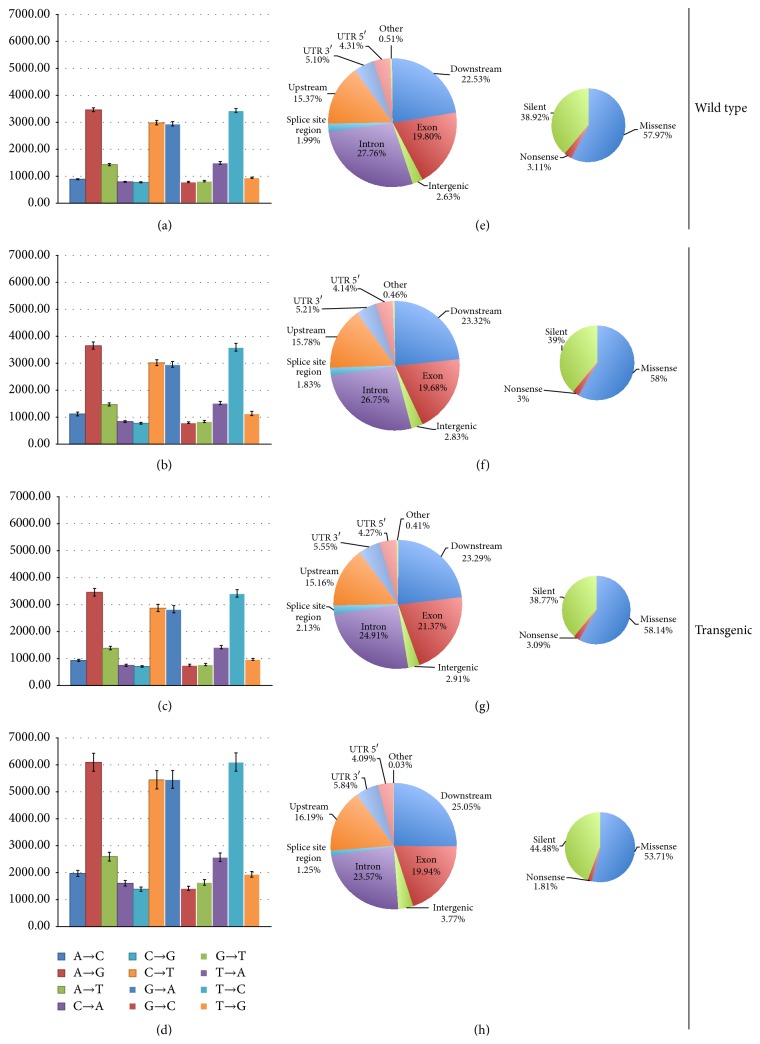
Polymorphism nucleotide base changes and predicted effects. Mean rates of change for each nucleotide base are shown for wild type (a), ST111 (b), ST77 (c), and 764 (d), with bars indicating the standard error of the mean. Regions containing detected polymorphisms and their functional classifications are shown for wild type (e), ST111 (f), ST77 (g), and 764 (h).

Total SNPs detected by samtools and bcftools across the wild type control group reported a mean of 20,707 SNPs per sample (Figure 1(a)), with a mean SNP rate of one polymorphism for every 42,561 nucleotides (Figure 1(b)). Of these, 48% on average are single allele polymorphisms with 35 multiallele sites containing 23 multiallelic SNPs. An overall transition (a purine base changed to another purine base or pyrimidine base changed to another pyrimidine base) to transversion (a purine base to pyrimidine base or pyrimidine base to purine base alteration) ratio (Ts/Tv) of 1.62 (Figure 1(d)) and an average of 1862 INDELS were identified per sample in the wild type group (Figure 1(c)). Mean base changes detected were as follows: 888 A→C, 3458 A→G, 1428 A→T, 793 C→A, 769 C→G, 2977 C→T, 2941 G→A, 787 G→C, 818 G→T, 1495 T→A, 3433 T→C, and 944 T→G (Figure 2(a)).

The ST111 experimental group reported an average of 20,208 SNPs per individual sample (Figure 1(a)), with a polymorphism rate of one SNP for every 44,323 nucleotides (Figure 1(b)). Similar to wild type, 48% of the SNPs in the ST111 line were single allele alterations, with an average of 34 multiallele sites and 25 multiallele SNPs. The Ts/Tv ratio was again very similar to wild type at 1.63 (Figure 1(d)) with an average of 1750 INDELS per sample (Figure 1(c)). Per base changes reported for the ST111 group were 933 A→C, 3457 A→G, 1382 A→T, 747 C→A, 710 C→G, 2868 C→T, 2830 G→A, 754 G→C, 779 G→T, 1420 T→A, 3415 T→C, and 968 T→G (Figure 2(b)).

The ST77 experimental group was also comparable to wild type with 21,666 average SNPs per sample (Figure 1(a)) at a rate of 41,225 nucleotides for every polymorphism (Figure 1(b)). Forty-nine percent of the detected SNPs were reported as singletons, with 33 identified multiallele SNP sites and 28 multiallele SNPs. The Ts/Tv ratio for the ST77 group was slightly lower than wild type at 1.56 (Figure 1(d)), indicating a slightly higher transversion rate in the ST77D and ST77F siblings. INDELS were nearly identical in number and size to the wild type control group with an average total of 1829 per sample (Figure 1(c)). Nucleotide base changes include 1119 A→C, 3650 A→G, 1468 A→T, 837 C→A, 772 C→G, 3015 C→T, 2952 G→A, 796 G→C, 842 G→T, 1520 T→A, 3589 T→C, and 1135 T→G (Figure 2(c)).

Lastly, the 764 experimental group contained more substantial nucleotide alterations when compared to wild type and the other two experimental groups (ST111 and ST77) revealing an average SNP count of 38,188 per individual (Figure 1(a)), nearly double the quantity of any other group. SNPs were also encountered on average at nearly double the frequency of the other samples, with one SNP recorded every 24,281 bases (Figure 1(b)). In this group, only 27% of SNPs were reported as singletons, with 78 multiallele sites and 44 multiallele SNPs. The Ts/Tv ratio was the lowest of all four groups at 1.53 (Figure 1(d)), indicating the 764 group had the highest overall rate of nucleotide transversions. INDELS also increased to 2390 with a larger deviation spread between samples (Figure 1(c)). Base changes included 1972 A→C, 6094 A→G, 2596 A→T, 1603 C→A, 1386 C→G, 5445 C→T, 5461 G→A, 1417 G→C, 1639 G→T, 2576 T→A, 6104 T→C, and 1940 T→G (Figure 2(d)).

Polymorphic sites unique to each transgenic event were identified by comparing each transgenic variant list to both WT and the remaining two transgenic groups. The ST77 transgenic group shared 2196 common gene variants with WT and exhibited 981 unique polymorphisms. The ST111 transgenic line revealed similar values sharing 1983 gene variants with the controls, while the 764 group shared 1912 variants. The ST111 and 764 lines each reported 927 and 7717 unique gene variants, respectively. Transgenic lines also revealed 127 gene variants shared between the three transgenic groups.

Overall, SNP calls clearly illustrated that the 764 experimental group contained significantly increased levels of polymorphisms over the wild type controls and other transgenic experimental groups, while concurrently demonstrating an increased quantity of more acute transversion type mutations. Average total SNPs, INDELS, SNP rates, and transition/transversion ratios for each experimental group are shown in Figure 1, and average individual nucleotide changes for each group are shown in Figures 2(a)–2(d).

### 3.2. Classification of Predicted SNP Effects and Impacts

Following SNP and INDEL detection with samtools, snpEff version 4.1i [31] was used to evaluate potential alterations to the exome resulting from these changes. SnpEff annotates variants based on their genomic locations, including introns, exons, upstream or downstream, splice sites, and untranslated regions at 5′ and 3′ sequence ends. Effects were grouped and sorted according to the variant type, potential level of effect impact, functional class, type, and region. No multinucleotide polymorphisms were detected in any individual of all four experimental groups, and modifications were limited to SNPs, insertions, and deletions of bases. Across all experimental groups, polymorphisms classified as low effect comprise ~10% of the total, while moderate effects and high effects average 12% and 2%, respectively. The largest category of impact classification is the genomic modifier category, comprising 72–78% of detected polymorphisms in all groups and 60% of shared variants between all transgenics (see snpEff reports in Supporting Data). Missense mutations are highly prevalent in all four groups, averaging ~60% in wild type, ST111, and ST77 events with a missense to silent mutation ratio of ~1.6 (Figures 2(e)–2(g)). The 764 group exhibited a slightly lower missense percentage of ~53%; however, it also contained a higher percentage of silent mutations at ~45% compared to the other three groups (~37%) generating a missense/silent mutation ratio of ~1.20 (Figure 2(h)). Nonsense mutations comprised ~3% of the total reported SNP effects for the wild type, ST111, and ST77 groups and ~1.8% in the 764 group (Figures 2(e)–2(h)).

All four experimental groups showed a high prevalence of SNPs of common types, namely, resulting in downstream gene variants (~24%), intron variants (~25%), missense variants (~11%), synonymous variants (~7%), and upstream gene variants (~16%). The most common locations for these variants are intron regions (~25%), the downstream transcription start site (~23%), exons (~23%), introns (~23%), and the upstream transcription start site (~16%). The 764 group distribution of polymorphism types and affected regions seem to mirror those of the wild type, ST111, and ST77 groups, with a slight reduction of SNPs in exonic regions. All individuals also reported variants within 5′ and 3′ untranslated regions (~4% and ~6%, resp.), as well as minimal detection of frameshifts, stop, and start variants (<1%). Regions and predicted effects of detected polymorphisms are shown in Figures 2(e)–2(h).

Transition base changes of A↔G and C↔T were most prevalent in all four experimental groups, while the transversion A↔T was ~50% more common than A↔C, C↔G, or G↔T transversions. Codon changes varied most commonly at the wobble base position, typically resulting from a transition in both wild type and transgenic samples. Following SNP patterns previously described, the prevailing modified codons appeared commonly shared between all individuals measured. Base changes in the first and second positions of the codon were much less frequent across all groups. Heat maps of the codon base changes representative for each experimental group are shown in Supplemental Figure 1. Predicted amino acid changes resulting from codon alterations predominantly consist of synonymous substitutions; however, other frequently altered residues include valine to alanine, alanine to valine, leucine to phenylalanine and proline, glutamate to glycine and lysine, serine to proline, and phenylalanine to leucine. Supplemental Figure 2 shows the distribution heat maps of detected amino acid substitution averages representative for all events.

Minor changes were also detected within the additional genomic scaffolds containing the transgene sequences supplemented to the alignment reference file. Specifically, snpEff reported SNPs in the transgenes of samples 764B3, 764H3, and 764K1 at position 80, ST77D2 at position 1400, ST77F1 at positions 500 and 700, ST77J3 at position 600, ST111B3 at position 800, ST111I3 at position 190, and ST111K1, ST111B1, and ST111K2 at position 1010. Read alignments were unable to verify all called variants in the transgenes, likely due to areas of low coverage; however, one area was corroborated as a consistent variant in the filler DNA of all constructs between the right border and promoter region (Supplemental Figure 3), which was included as a control. Supplemental Table 1 summarizes all SNP calls and between group statistical tests. Summaries of all SNP/INDEL calls from bcftools and their respective effects from snpEff, along with chromosomal distribution plots, additional statistics, and lists of SNPs shared between groups and within each group, are available from the iPlant collaborative Discovery Environment [33]. Links to the respective datasets are provided in the Supporting Data of this manuscript.

### 3.3. Gene Ontology Analysis of Genes Containing SNPs

**Figure 3:**
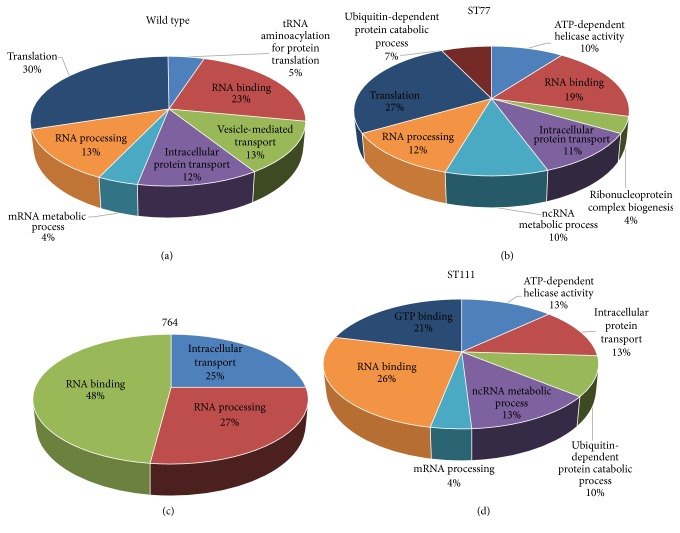
AgriGO enriched gene ontology categories of SNP-containing genes. Single enrichment analyses for all genes containing SNPs are shown for wild type (a), ST77 (b), 764 (c), and ST111 (d). Percentages are reflective of how many terms matched the indicated GO group out of the total matches to the background reference.

**Figure 4:**
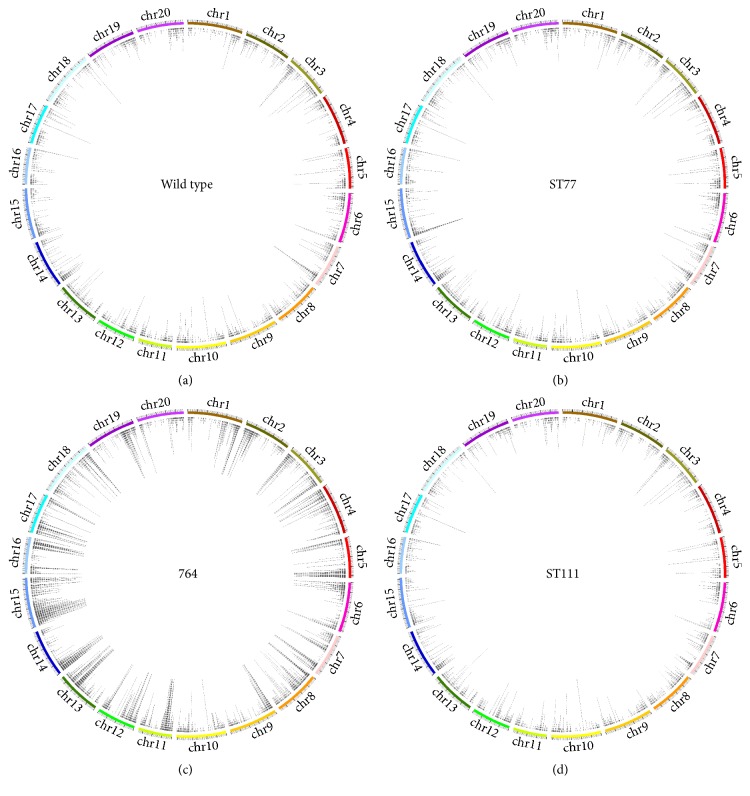
Overview of the chromosomal distribution of detected transcript SNPs. Aligned SNPs are shown for each soybean chromosome in the wild type controls (a), ST77 (b), 764 (c), and ST111 (d) transgenics. The colored bars indicate each chromosome scaled to length, with a bold outer hash mark representing every 10 Mb of sequence and each nonbolded outer hash mark placed every 1 Mb. Each internal tick represents a detected SNP, on which subsequent SNPs are stacked vertically from each transcript at that region.

Given the extent of detected polymorphic sites and the possibilities of effectual contributions to gene expression changes previously documented in the 764 transgenic line, it was necessary to investigate genes containing SNPs and INDELS with gene ontology to identify the possible functional pathways controlled by the affected transcripts. Complete gene lists containing SNPs provided by the output of snpEff for the wild type (36,959 transcripts), ST77 (38,691 transcripts), ST111 (34,142 transcripts), and 764 (43,426 transcripts) lines were loaded into the AgriGO online gene ontology analysis tool and subjected to a single enrichment analysis with multiple corrections to deduce the functional roles of polymorphic genes. Out of the total* Glycine maxV2.1* background set of 29,501 GO terms, wild type matched 11,245, ST77 matched 11,660, ST111 matched 10,326, and 764 matched 13,321. GO categories were similar between the wild type, ST77, and ST111 groups with minor variations, with the majority of terms putatively annotated to translational or RNA processes (Figures 3(a), 3(b), and 3(d)). Intracellular transport also constituted a large portion of the identified categories (~12% of total annotations) and, in the case of the 764 transgenic line, was the only other significant GO term following RNA processing and binding (Figure 3(c)). Three categories including ATP-dependent helicase activity, ubiquitin-dependent protein catabolic processes, and ncRNA metabolic processes were all unique to the ST77 and ST111 lines (Figures 3(b) and 3(d)). GTP binding was exclusive to the ST111 line, comprising 21% of the total significant GO terms for the event.  Figure 4 shows all detected SNPs in a chromosomal density map for all four experimental groups.

Annotated GO terms for SNPs exclusive to each transgenic event displayed similar functions, with ST77 reporting 295 gene variants involved with intracellular organelles, photosystem II, cytoskeletal components, and protein complexes. ST111 displayed similar numbers of terms totaling 273 annotations, including photosynthesis and photosystem II, intracellular organelles, ribonucleoprotein complexes, thylakoids, protein complexes, and oxidoreductase activity. The 764 line exhibited the highest number of unique annotated variants with 2366 involving translation, gene expression, biosynthetic processes, ribosomal structures, and protein complexes. Of the 127 polymorphic genes shared by all transgenic specimens, 40 annotations returned 10 GO terms pertaining to intracellular organelles, macromolecular complexes, and protein complexes. Specific GO terms for each group are shown in Figure 3, and complete AgriGO feature relationship trees are shown in Supplemental Figure 4.

## 4. Discussion

SNP rates have been previously evaluated in soybean using multiple fragment analysis on several genotypes of cultivated varieties, including Lincoln, Mandarin, Peking, and Richland [34, 35]. From this fragment analysis, transition and transversion rates were reported to be nearly identical between cultivars at 48% and 52%, respectively. Furthermore, nucleotide diversity rates of cultivated soybean varieties were estimated to be 5–8-fold less than the wild variety* Glycine soja* and occur at an even lower frequency than the highly characterized* Arabidopsis thaliana* self-crossing model [34]. More recent investigations of SNPs and INDELS in soybean using next generation resequencing have revealed genome-wide polymorphisms in an effort to identify native disease resistant and favorable trait loci [36, 37]. Further studies demonstrate the extensive narrowing of soybean genomic variation due to selective pressures resulting from domestication practices which favor more valued traits, such as increased seed mass and oil content quantitative trait loci [38]. In addition, the self-pollinating nature of* Glycine max* also contributes evolutionarily to the constriction of genomic variances in domesticated cultivars. Thus, with such selective pressures bottlenecking natural variations in cultivated soybean varieties, it is quite intriguing to see such consistently increased numbers of SNPs in only one transgenic experimental group with no definitive initiating event.

SNPs detected in our datasets were divulged from seed transcriptome sequences, which represent ~6.5% of the total soybean genome and 65% of total genomic protein coding sequences. The SNPs described here are not considered to be representative of the frequencies that may be detected in other soybean tissues, or in different developmental stages of these specific transgenic plants; rather, they demonstrate increased transcript polymorphism rates witnessed in a single soybean transformant out of several different experimental groups. Although RNA-seq is focused on the functional segments of the genome and does not always capture regulatory regions such as promoters or nontranscribed regions (e.g., methylated bases), one aspect of the resulting effects of polymorphisms in these regions can be directly witnessed through examination of gene expression levels. Because the soybean expression system described previously by our lab [5, 7, 8, 13, 14, 39] specifically targets seed tissue for recombinant protein accumulation, it was of great interest to identify possible sequence alterations that may have resulted in gene expression or protein structure variations in seeds, which to date has not been investigated.

Base changes for wild type, ST77, and ST111 events were all comparable, while the 764 event consistently reported SNP rates nearly double that of the other groups (1 SNP detected every ~22,000 bases), which is still well below the previously reported SNP rate of 1 SNP per ~1,400 nucleotides in soybean seeds [40]. Nevertheless, SNP base changes appeared to follow the same pattern of commonality, with all groups demonstrating high percentages of transition base changes with relatively low transversion counts. The 764 line did demonstrate a lower Ts/Tv ratio than the other groups (Figure 1(d)), although progeny from two independent parents in the ST77 line (ST77D and ST77F) exhibited lower ratios as well, indicating that this characteristic alone can not reliably indicate internal stresses or overall divergence from our controls. The majority of detected base changes were located in the third base of the codon, likely generating synonymous (silent) polymorphisms which were the majority of predicted SNP classifications in all events. Less common changes occurred in the first or second bases of the codon, generating a nonsynonymous or missense mutation likely resulting in an amino acid change (Figures 2(e)–2(h)). Missense to silent ratios detected in all experimental groups appear to be higher than previously published results for soybean indicated [40], although the higher nonsynonymous to synonymous mutations may be due to high linkage disequilibrium exhibited in soy [41].

Interestingly, the 764 line had the lowest overall missense to silent polymorphism ratio, demonstrating that while the 764 line contained the highest overall number of SNPs, a higher percentage of the total polymorphisms were silent mutations (~45%) compared to the other three experimental groups (Figure 2(h)). This reveals the possibility that SNPs and INDELS detected here had originated from the original transformation event and have been highly conserved through self-crossed generations of progeny, particularly considering how remarkably well conserved the attributes of detected SNPs were between different biological samples of the same transformation event. Without sequencing data from prior generations of each parental line tested here for comparison, this can not be confirmed with complete confidence; however, this does pose an interesting inquiry that even silent SNPs may have the ability to alter gene expression and peptide structure. Indeed, it has been suggested that although synonymous polymorphisms code for identical amino acids, subtle changes resulting from the utilization of nonoptimal synonymous codons can modulate downstream transcription and translation rates [42], thereby altering the folding conformations and proposed function of the final peptides. This may explain why the 764 transgenic event exhibited the highest rates of differentially expressed genes in conjunction with polymorphisms but still maintained a healthy phenotype.

While many SNP studies have excluded synonymous mutations and those present in noncoding regions, recent works have examined these sequences in light of their possible epigenetic effects on transcription and translation; however, more advanced tools for the prediction of possible effects are still in development and require further testing for reliable forecasts [43]. Nonsynonymous polymorphisms appear at similar rates across the experimental groups, varying between 50 and 60% of total functional SNP classifications. While nonsynonymous mutations nearly always alter amino acid sequences that can potentially yield nonfunctional protein products, this phenomenon may produce a neutral effect on the organism or protein or activate signaling cascades in redundant systems [43]. The effects of these detected nonsynonymous polymorphisms in each transgenic event are not known or identifiable without extensive proteomic investigations into the altered downstream transcript products. The 764 line, which previously displayed the largest degree of differential gene expression, exhibited the lowest percentage of missense and nonsense polymorphisms; therefore, these alone are not likely to be the root cause of observed changes in this transgenic line.

Spontaneous mutations may also occur as a result of a previously occurring stressful event, such as parental line* Agrobacterium* transformation. Epigenetic alterations and nucleotide transpositions have been detected up to five generations forward from transformation in* Arabidopsis* [44, 45]; however, all seeds examined here were bred to or beyond the 5th generation of progeny. The causes for these mutations are unknown; however, each transformant was an independent transformation event and was likely exposed to a varying degree of stress during the process. This could potentially have introduced genomic variations that have been carried forward to the transcriptome, as even large copy number of variations have been reported in plants as a response to stress [46]. Segregation of SNPs from existing parental heterozygous loci is expected in progeny; however, all samples examined here were remarkably consistent in SNP numbers, location distributions, and types within their experimental groups, further demonstrating the genomic stability of these events. This further solidifies the possibility that the detected variations in all transgenic lines arose from an early parental event, possibly occurring during the original* Agrobacterium* transformation or planting event, and have been stably integrated and carried forward to the generations examined. Considering the consistency of the SNP patterns, these polymorphisms have likely originated at the genomic level and are not likely to appear in such large quantities due to transcriptional proofreading errors.

Detected alterations occurred in a “bowtie”-shaped distribution across the 20 chromosomes, with lower instances of SNPs near the centromeres and the highest incidence of SNPs near the euchromatic telomeric ends of the chromosomal arms. This is where gene density is the highest, which has been illustrated previously by several works conducted on soybean polymorphism rates [36, 38, 47]. Our snpEff dataset summaries available in the Supporting Data of this manuscript show the same distribution pattern, with no significant alterations of SNP rates between biological replicates of progeny. Without complete characterization of the targeted SNPs in this work, future directions can invoke functional characterizations to cross-reference detected polymorphisms with possible connections to differentially expressed genes and also to improve existing annotated Williams 82 cultivar SNPs. In order to fully deduce the origin of SNP variations with high confidence, multiple generation analyses will need to be conducted. Approximately 70% of the genes that were differentially expressed also contained SNPs or INDELS; however, due to the extreme disparity in size between each gene list (a maximum of ~3,000 and ~40,000 genes, resp.), this could simply be due to chance and can not definitively hold concrete biological significance without further investigation. This suggests that the SNPs examined here may directly impact native gene expression levels, possibly due to their locations within gene regulatory regions or enhancer elements located near the telomeric ends of the chromosomal arms.

Gene ontology terms from SNP gene lists of all experimental groups were very similar, returning many enriched terms regarding protein transport and localization as well as RNA and transcriptional processes even in the wild type group (Figure 3 and Supplemental Figure 4). Interestingly, these relationships are highly similar to the enriched GO terms derived from the differentially expressed gene sets of the transgenic lines, indicating the majority of functional relationships between these polymorphisms are likely arising from intercultivar variations and are not specifically related to transgenesis effects. However, given the possibility that mutations from transgenesis could occur within QTLs that control traits such as plant size, flower count, and seed quantity, identification of novel polymorphisms in these regions of soybean's transcriptome is of great importance to both plant breeders and in biotechnology applications. In fact, recent investigations have revealed an excess of 9 million unique SNPs that occur between 106 different cultivated soybean varieties [48]. Here, we have provided lists of exonic SNPs unique to each of the three transgenic groups and specific to transformation, as well as novel SNPs detected in our Williams 82 wild type controls. Further investigations of the events described will identify the sites of T-DNA integration and characterize the surrounding sequences to determine whether increased incidences of polymorphisms occur within the immediate vicinities of the insertions. Unique SNPs identified and reported here may be used to aide these investigations of crucial trait loci in the future to maximize yields and assist in breeding programs of transgenic specimens.

## 5. Conclusions

This study describes the first direct comparison of polymorphism rates present in the seed transcriptome of three independent transgenic lines of* Glycine max* with a nontransgenic wild type line, all derived from the Williams 82 soybean cultivar. Previously, we examined gene expression changes in these transgenic events, finding only the 764 line to be significantly altered when compared to the other transgenic lines. In the same manner, we have expanded our previous work and reported transcriptome single nucleotide polymorphism rates for the ST77, ST111, and 764 transgenic events compared to wild type. Mirroring the results from our differential expression study, the samples of the 764 transformation event exhibited transcript SNP rates nearly double that of the wild type, ST77, and ST111 events, while also demonstrating higher amounts of transversion mutations. While the exact cause for both the large number of differentially expressed genes and polymorphisms present in the 764 event is not known, it is highly likely that the two phenomena are correlated. Our results show that, in addition to differential expression, transgenesis may result in unpredicted base changes within gene transcripts and regulatory regions without impacting plant phenotype.

## Availability of Supporting Data

The RNA sequencing data described herein may be accessed at http://www.ncbi.nlm.nih.gov/geo/query/acc.cgi?acc=GSE64620 through NCBI's Gene Expression Omnibus under accession number GSE64620. Summary files for bcftools and snpEff outputs encompassing variant calls and predicted variant effects are available from the iPlant Collaborative Discovery Environment directory available at http://de.iplantcollaborative.org/dl/d/44044BEE-69B8-4129-A6B5-4C9291B6B4A1/snpeffsummaries.zip and http://de.iplantcollaborative.org/dl/d/AD464040-9169-4E52-87D5-DEF7466A2C1E/variantsummaries.zip. Lists of genes containing detected variants from all samples are available from iPlant in a compressed  .zip archive available at http://de.iplantcollaborative.org/dl/d/533570A3-1EFB-4864-B9A9-9D82F17E09A8/snpeffgenes.zip.

## Supplementary Material

Supplemental Figure 1. Heatmap of detected codon changes across wild type and all three transgenic groups. Green color denotes less frequent occurrence, and red denotes a higher occurrence.
Supplemental Figure 2. Amino acid change heatmap for wild type and the three transgenic groups. Darker colors represent a higher detected instance of the respective amino acid alteration.
Supplemental Figure 3. Base change controls in transgenes. Variants located at bases 232 and 233 were located in the padded sequence after the right border, and were detected consistently in all events with transcripts across this region, demonstrating the repeatability and consistency of the SNP calls.
Supplemental Figure 4. AgriGO single enrichment analysis results of the genes containing effectual SNPs in transgenic events. Darker colors indicate a higher level of significance for each node.
Supplemental Table 1. Raw SNP, base changes and deviation values for each sample.
