# Supplementary material for: Transcript Polymorphism Rates in Soybean Seed Tissue Are Increased in a Single Transformant of Glycine max
Source: Int J Plant Genomics. 2016 Nov 29;2016:1562041. doi: 10.1155/2016/1562041 (PMC5153505; doi:10.1155/2016/1562041)
Supplement: Supplementary file 1 — Supplemental Figure 1. Heatmap of detected codon changes across wild type and all three transgenic groups. Green color denotes less frequent occurrence, and red denotes a higher occurrence. Supplemental Figure 2. Amino acid change heatmap for wild type and the three transgenic groups. Darker colors represent a higher detected instance of the respective amino acid alteration. Supplemental Figure 3. Base change controls in transgenes. Variants located at bases 232 and 233 were located in the padded sequence after the right border, and were detected consistently in all events with transcripts across this region, demonstrating the repeatability and consistency of the SNP calls. Supplemental Figure 4. AgriGO single enrichment analysis results of the genes containing effectual SNPs in transgenic events. Darker colors indicate a higher level of significance for each node. Supplemental Table 1. Raw SNP, base changes and deviation values for each sample. [file 1562041.f1.pdf]

Supplemental Figure 1.

Wild Type

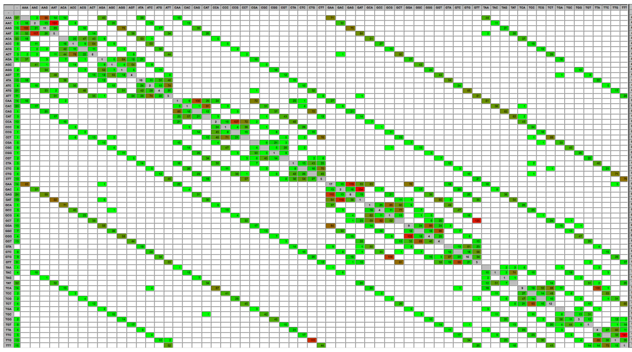

ST111

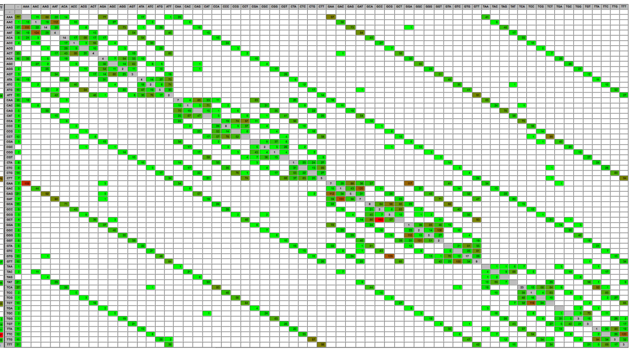

ST77

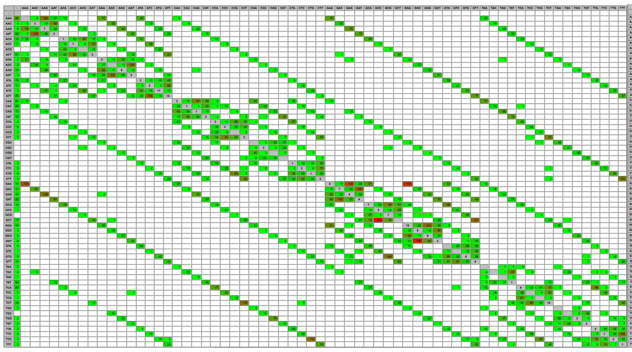

764

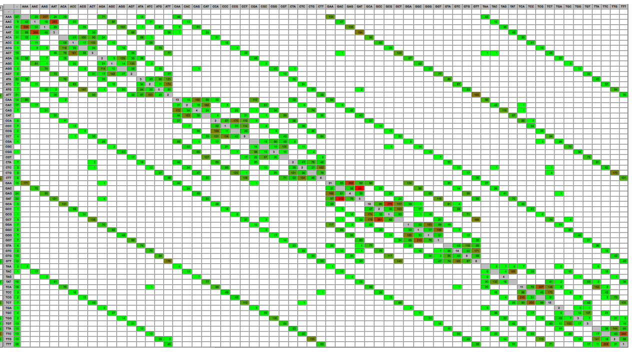

Supplemental Figure 2.

Wild Type

	*	-	?	A	C	D	E	F	G	H	I	K	L	M	N	P	Q	R	S	T	V	W	Y
*	9	3					1		5			1	4				8	5	6			4	3
-			354																				
?																							
A		15		573	1	26	42		53							58			76	173	237		
C	10	6			76			14	17	1			1					57	63		1	22	24
D		20		18		255	131		83	34					119			6			58		63
E	54	38		84		95	254		114			142	3				53	1			38		
F		20			19			215			56		137						66		42		25
G	15	34		54	39	60	99		616									154	72		31	48	
H		23				20				149			16		34	6	50	75	3				120
I		30				1		29			278	18	53	63	41			13	36	103	139		
K	65	47					146				26	211		17	60		48	118		24			
L	29	44						190		44	72		816	22		135	39	27	62		91	4	
M		21									90	33	49	4	2			32		48	65		
N	2	12				96				38	47	87			255				63	22			37
P		21		38						15			169			467	35	16	154	36			
Q	123	15					48			66		36	96			27	181	109		2			
R	49	27		1	62				80	48	21	74	10	11		57	38	348	45	15		37	
S	36	29		56	24			120	84		19	1	131		89	182		66	560	61	2	6	19
T		40		151		1					145	22		30	26	14		10	114	411			
V		26		271	2	25	28	32	72		99		89	34							464		
W	61	2			23			2	10				12					59	2			3	
Y	31	36			50	26		28		49			2		42				24				126

ST111

	*	-	?	A	C	D	E	F	G	H	I	K	L	M	N	P	Q	R	S	T	V	W	Y
*	8	3							8			4	2				7	6	7			13	9
-			386																				
?																							
A		31		761	1	24	26		44							100		2	63	162	254		
C	6	9			118			28	9	1								50	59		1	30	59
D		10		34		259	128		102	27					134			1			61		35
E	63	28		63		121	212		163			251	2				91	6			73		
F		43			27			192			46		181						88		50		22
G	14	26		94	48	65	101		652									101	85		48	45	
H		18				65				139			32		23	11	85	73	1				62
I		27						39			298	13	43	60	44			32	12	115	169		1
K	87	94					170		1		19	213		30	75		41	150		22			
L	30	56						229		38	62		796	37		219	36	90	74		129	6	
M		15				1					96	27	49	5	2			22		84	41		
N		35				122				21	50	82			241				81	23			25
P		28		40						19			140			521	47	23	224	21			
Q	141	21					34			71		48	47			18	162	98		4			
R	66	23		1	47				77	75	12	63	13	10		11	52	373	74	31		40	
S	26	53		88	50			128	106		27	2	119	5	61	246	1	115	655	78	1	3	17
T		42		130		1					147	22		25	30	44		20	78	400			
V		32		293	1	30	46	41	76		107	1	104	48							541		
W	57	1			20			2	19				28					82	6			3	
Y	30	23			52	16		26		51			1		40				34				127

ST77

	*	-	?	A	C	D	E	F	G	H	I	K	L	M	N	P	Q	R	S	T	V	W	Y
*	5	5					2		5				2				7	9	7			4	9
-			402																				
?																							
A		28		714	1	42	35		59							70			62	136	307		
C	14	5			102			9	17									35	60			14	16
D		37		27		244	173		112	46					151						80		43
E	36	20		91		105	209		216			239					107	4			95		
F		10			34			177			41		148						84		68		10
G	26	31		65	36	74	118		781									105	68		46	43	
H		35				7				124			26		25	30	75	126	1				100
I		50						73			304	16	50	39	24			21	20	98	137		
K	48	74					157				62	194		33	76		30	136		27			
L	50	23			1			221		35	53		791	35		246	42	71	109		122	18	
M		4									96	47	65	17	1			33		62	51		
N		39				108				24	52	66			220				117	14			42
P		24		38						16			156			471	42	33	191	46			
Q	143	34					31			124		31	44			12	166	88		2			
R	50	21		1	78		2		67	66	11	103	8	13		24	36	432	64	12		43	
S	25	55		75	46			116	73		17	3	111		65	260		86	721	62	1	4	36
T		51		127		1					191	35		30	26	35		11	93	418			
V		22		257	6	29	38	35	89		84		73	45							517		
W	81	2			21			3	24				14					104	1			3	
Y	26	28			65	32		26		62			3		32				21				130

764

	*	-	?	A	C	D	E	F	G	H	I	K	L	M	N	P	Q	R	S	T	V	W	Y
*	30	3			1		5		4			4	6				11	5	7			4	8
-			415																				
?																							
A		8		1,555	4	28	66		101							89		2	163	356	393		
C	19	17			263			36	26									93	118			35	78
D		26		46		668	270		217	78					176			4			70		101
E	69	34		110		234	475		193			266					93	2			134		
F		41			40			453			55		209						104		91		53
G	19	27		94	43	125	178		1,199									171	164		70	45	
H		17				52				325			35		52	30	119	151	1				129
I		50						70			605	35	148	123	41			25	48	231	348		
K	77	58					269				15	443		51	116		111	173		54			
L	34	47						345		57	94		1,660	72		359	91	124	150	3	154	18	1
M		7				2					134	40	121	5	2			31		147	53		
N		21				231				36	49	173			535		1		174	50			61
P		19		80						36			194			1,113	67	66	225	60			
Q	217	21					106			142		91	124			45	355	167	1	2			
R	50	22		1	143				143	167	28	136	32	18		62	132	939	113	36		47	
S	34	37		174	89			149	139		41	1	227		153	299		101	1,686	191	1	7	41
T	1	37		306		1					202	37		73	43	63		42	188	925			1
V		38		415		45	60	57	122		325		201	84							1,026		
W	81	2			14			2	31				21					119	15			3	
Y	48	17			68	37		49		92			2		58				66				321

Supplemental Figure 3.

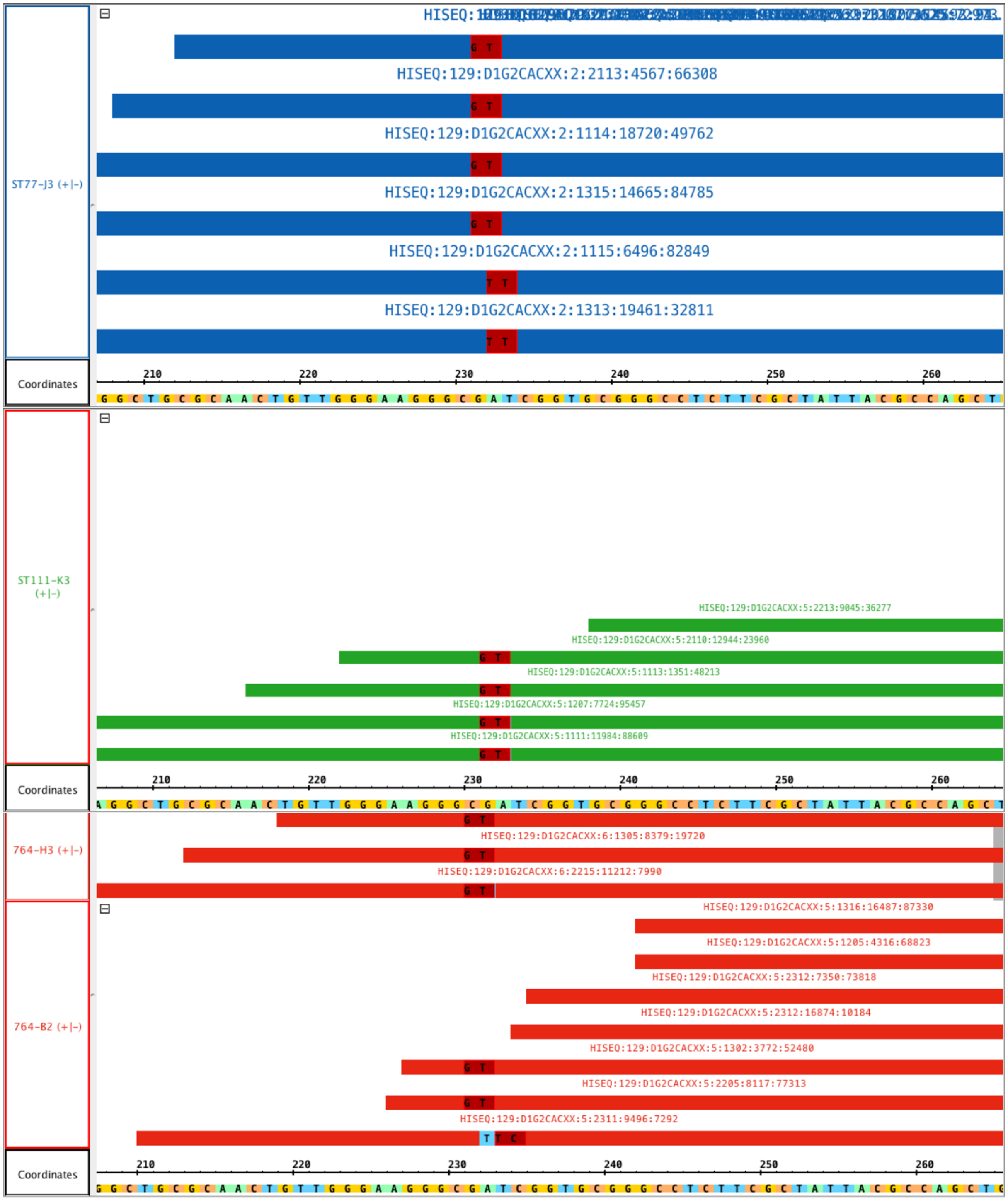

Supplemental Figure 4.

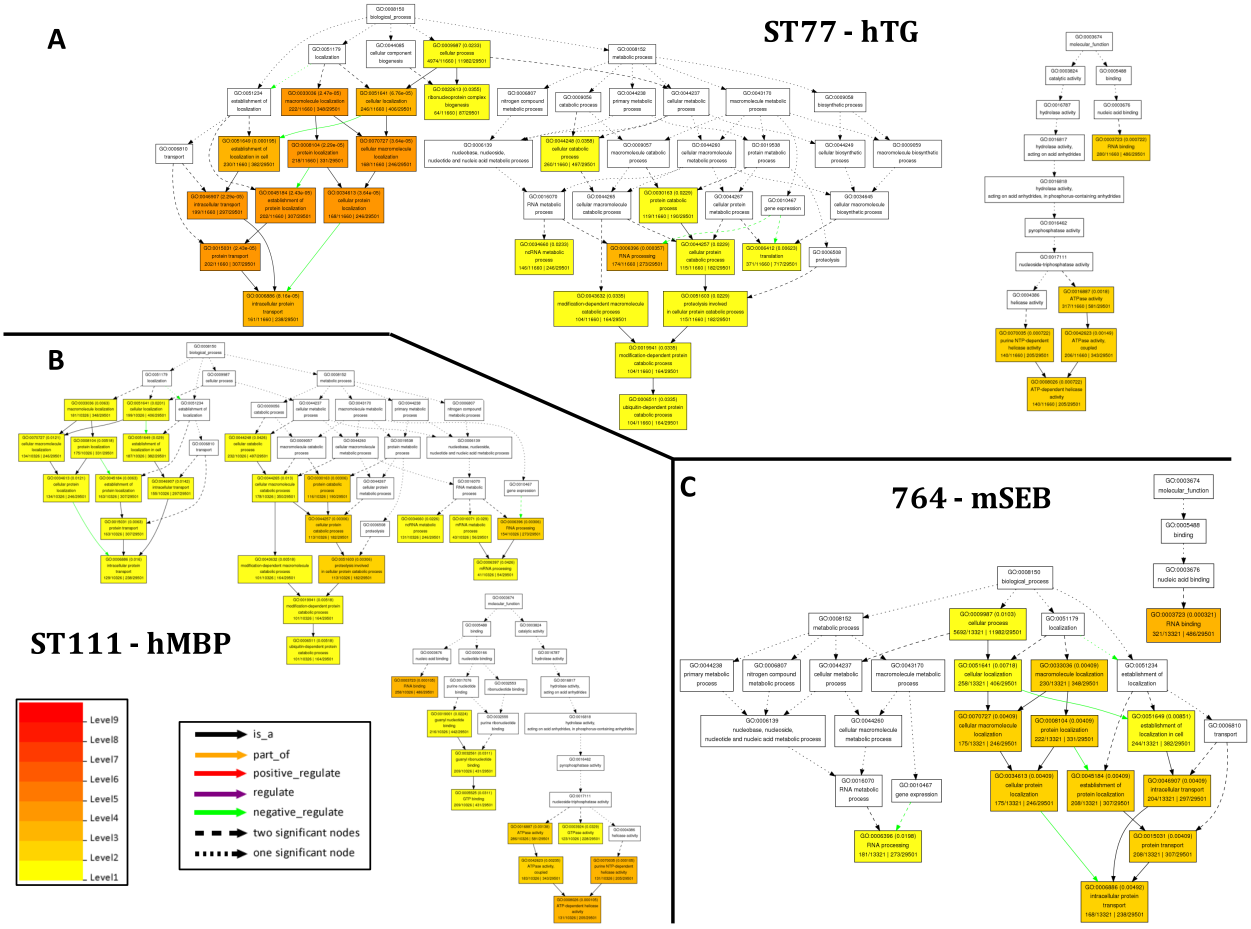

Supplemental Table 1.

Sample	SNPs	TS/TV Ratio	Indels	singleton snps	singleton ts/tv	singleton indels	multiallele sites	multiallele snps	SNP Rate AVG	A>C	A>G	A>T	C>A	C>G	C>T	G>A	G>C	G>T	T>A	T>C	T>G
WTB1	21096	1.61	1964	50.80%	1.7	57.00%	37	23	41479	889	3609	1497	811	782	3005	2973	816	814	1545	3442	936
WTB2	20388	1.63	1914	48.70%	1.72	57.01%	34	24	42874	859	3412	1435	794	727	2978	2883	748	818	1509	3377	872
WTB3	23047	1.61	2225	52.20%	1.68	58.30%	44	27	37826	972	3827	1661	825	840	3271	3348	876	902	1756	3782	1014
WTH1	21650	1.62	1957	48.10%	1.67	57.70%	43	25	40496	932	3464	1448	843	833	3194	3160	806	891	1530	3576	998
WTH2	20605	1.57	1786	46.70%	1.65	55.20%	31	19	42715	902	3361	1417	813	759	2972	2903	808	876	1493	3377	943
WTH3	21760	1.6	1928	46.20%	1.7	55.70%	41	29	40362	931	3601	1513	828	793	3139	3057	817	846	1612	3620	1032
WTL1	18643	1.65	1527	46.30%	1.74	55.10%	27	13	47416	820	3194	1230	747	699	2561	2607	725	697	1233	3245	898
WTL2	18640	1.6	1622	47.20%	1.72	56.40%	31	26	47179	823	3165	1245	716	709	2642	2603	718	723	1328	3088	906
WTL3	20536	1.67	1843	47.50%	1.74	55.80%	30	22	42704	864	3493	1404	757	776	3031	2938	767	794	1449	3391	894
**Mean**	20707.22	1.62	1862.89	0.48	1.70	0.56	35.33	23.11	42561.22	888.00	3458.44	1427.78	792.67	768.67	2977.00	2941.33	786.78	817.89	1495.00	3433.11	943.67
**StdDev**	1427.37	0.03	204.39	0.02	0.03	0.01	6.18	4.78	3117.09	51.60	208.49	132.34	43.03	50.37	236.85	239.45	51.22	71.41	151.82	206.07	57.95
**StdErr**	475.79	0.01	68.13	0.01	0.01	0.00	2.06	1.59	1039.03	17.20	69.50	44.11	14.34	16.79	78.95	79.82	17.07	23.80	50.61	68.69	19.32
ST111B1	21301	1.64	1984	49.40%	1.67	56.70%	40	26	41064	961	3577	1467	812	739	3121	2968	833	803	1500	3569	978
ST111B2	22297	1.61	1997	49.20%	1.68	59.20%	44	34	39331	1034	3676	1553	840	787	3228	3172	808	882	1562	3714	1076
ST111B3	20363	1.64	1909	49.00%	1.71	57.20%	40	30	42417	911	3487	1470	767	743	2970	2959	772	828	1386	3434	940
ST111I1	19005	1.65	1510	44.70%	1.77	56.90%	26	21	46636	877	3253	1271	705	667	2739	2724	689	724	1348	3143	888
ST111I2	18356	1.64	1530	45.70%	1.79	54.50%	32	26	48097	821	3185	1266	671	662	2556	2592	684	662	1328	3079	876
ST111I3	21702	1.66	1981	44.70%	1.68	56.00%	38	29	40444	997	3899	1471	788	715	2911	2952	779	857	1556	3805	1002
ST111K1	19709	1.63	1450	47.20%	1.73	56.10%	24	20	45232	965	3386	1355	706	715	2661	2688	731	709	1334	3487	993
ST111K2	24097	1.62	2326	52.90%	1.68	59.10%	45	26	36242	1095	4056	1596	898	827	3534	3436	931	984	1703	3907	1156
ST111K3	15044	1.6	1065	45.40%	1.69	56.50%	14	11	59443	732	2597	996	540	538	2090	1976	562	563	1065	2597	800
**Mean**	20208.22	1.63	1750.22	0.48	1.71	0.57	33.67	24.78	44322.89	932.56	3457.33	1382.78	747.44	710.33	2867.78	2829.67	754.33	779.11	1420.22	3415.00	967.67
**StdDev**	2615.63	0.02	385.91	0.03	0.04	0.01	10.46	6.72	6787.46	111.19	429.78	184.87	105.96	83.17	420.05	411.12	104.85	127.46	184.86	413.65	107.68
**StdErr**	871.88	0.01	128.64	0.01	0.01	0.00	3.49	2.24	2262.49	37.06	143.26	61.62	35.32	27.72	140.02	137.04	34.95	42.49	61.62	137.88	35.89
ST77D1	23491	1.5	2145	51.60%	1.54	58.40%	45	32	37285	1305	3891	1640	897	821	3304	3084	860	903	1544	3841	1434
ST77D2	22880	1.52	2016	49.80%	1.55	58.40%	46	29	38410	1282	3898	1531	845	808	3070	2974	808	899	1605	3894	1295
ST77D3	24214	1.51	2202	52.50%	1.55	56.20%	43	28	36220	1345	4058	1653	964	859	3323	3242	877	957	1703	3959	1304
ST77F1	18610	1.51	1396	46.00%	1.56	56.40%	28	21	47788	1045	3125	1244	740	644	2569	2476	692	729	1279	3029	1061
ST77F2	20530	1.51	1624	49.50%	1.56	57.80%	26	16	43163	1112	3446	1406	798	701	2789	2748	784	809	1482	3388	1085
ST77F3	24879	1.51	2110	50.10%	1.56	56.50%	52	37	35419	1362	4066	1688	948	919	3421	3369	915	983	1752	4130	1365
ST77J1	18520	1.64	1519	48.90%	1.76	54.90%	23	19	47715	806	3150	1257	707	688	2655	2660	699	714	1358	3057	791
ST77J2	22737	1.68	1906	47.10%	1.74	58.20%	45	31	38769	969	3923	1496	865	823	3244	3279	818	890	1619	3841	1003
ST77J3	19129	1.66	1540	49.40%	1.76	58.60%	33	28	46259	842	3292	1300	770	681	2758	2735	708	696	1342	3159	876
**Mean**	21665.56	1.56	1828.67	0.49	1.62	0.57	37.89	26.78	41225.33	1118.67	3649.89	1468.33	837.11	771.56	3014.78	2951.89	795.67	842.22	1520.44	3588.67	1134.89
**StdDev**	2493.25	0.08	309.83	0.02	0.10	0.01	10.47	6.78	5031.52	216.22	391.96	174.54	90.29	95.02	324.87	312.92	81.82	108.36	166.72	428.88	225.63
**StdErr**	831.08	0.03	103.28	0.01	0.03	0.00	3.49	2.26	1677.17	72.07	130.65	58.18	30.10	31.67	108.29	104.31	27.27	36.12	55.57	142.96	75.21
764B1	46578	1.55	3098	26.10%	1.67	41.90%	69	40	19267	2256	7393	3220	2012	1625	6736	6737	1662	2036	3198	7502	2241
764B2	26386	1.54	1566	27.30%	1.68	41.60%	56	32	34217	1345	4318	1752	1075	980	3677	3729	999	1109	1784	4309	1342
764B3	40747	1.55	2662	26.20%	1.67	43.40%	83	34	22032	1958	6508	2792	1694	1465	5922	5820	1582	1780	2755	6513	1992
764H1	35176	1.5	2144	26.50%	1.55	41.80%	67	39	25360	1835	5588	2383	1501	1356	4924	5009	1327	1494	2360	5627	1813
764H2	44328	1.49	2869	26.20%	1.56	41.60%	104	55	20267	2303	6989	3053	1904	1630	6385	6321	1646	1908	3019	6890	2336
764H3	35356	1.53	2027	26.60%	1.58	41.10%	86	56	25572	1948	5795	2379	1455	1241	4941	4953	1328	1473	2389	5724	1787
764K1	44693	1.51	2889	26.80%	1.61	42.80%	91	52	20090	2366	7049	3021	1872	1627	6360	6419	1634	1955	3066	7110	2267
764K2	31736	1.54	1855	27.90%	1.62	42.70%	68	45	28445	1712	5070	2119	1302	1122	4505	4539	1189	1330	2045	5161	1687
764K3	38690	1.53	2400	25.40%	1.6	40.90%	82	45	23277	2022	6132	2643	1615	1431	5552	5624	1390	1666	2566	6097	1998
**Mean**	38187.78	1.53	2390.00	0.27	1.62	0.42	78.44	44.22	24280.78	1971.67	6093.56	2595.78	1603.33	1386.33	5444.67	5461.22	1417.44	1639.00	2575.78	6103.67	1940.33
**StdDev**	6663.47	0.02	525.75	0.01	0.05	0.01	14.72	8.77	4792.92	321.76	1007.56	481.12	304.21	234.38	1010.03	984.46	231.90	311.33	480.39	1014.57	320.46
**StdErr**	2221.16	0.01	175.25	0.00	0.02	0.00	4.91	2.92	1597.64	107.25	335.85	160.37	101.40	78.13	336.68	328.15	77.30	103.78	160.13	338.19	106.82
